# Comparison of refractive outcomes after photorefractive keratectomy with different optical zones using Mel 90 excimer laser

**DOI:** 10.1186/s12886-020-01537-3

**Published:** 2020-07-09

**Authors:** Dae Hwan Shin, Yong Woo Lee, Ji Eun Song, Chul Young Choi

**Affiliations:** 1 Department of Ophthalmology, Kangbuk Samsung Hospital, Sungkyunkwan University School of Medicine, 29 Saemunan-ro, Jongno-gu, Seoul, 03181 Republic of Korea; 2 Kim’s Eye Hospital, Seoul, Republic of Korea

**Keywords:** Photorefractive keratectomy, Optical zone, Myopic regression, Mel 90 excimer laser

## Abstract

**Background:**

A larger optical zone for photorefractive keratectomy may improve optical quality and stability. However, there is need for limiting ablation diameter in that a larger ablation diameter requires greater ablation depth, and minimizing ablation depth may reduce adverse effects on postoperative wound healing, haze and keratoectasia. In this study, we compared the changes in clinical outcomes and the degree of regression between a 6.0 mm optical zone and 6.5 mm optical zone following PRK.

**Methods:**

The records of 95 eyes that had undergone PRK with a 6.0 OZ (*n* = 40) and a 6.5 OZ (*n* = 55) were retrospectively reviewed. We compared data including the spherical equivalent of manifest refraction (SE of MR), simulated K (Sim K), thinnest corneal thickness, change in thinnest corneal thickness (the initial value divided by corrected diopter [ΔTCT/CD]), Q value, corneal higher order aberrations (HOAs) and spherical aberration (SA) pre-operation, at 3 and 6 months postoperative and at the last follow-up visit (Mean; 20.71 ± 10.52, 17.47 ± 6.57 months in the 6.0 and 6.5 OZ group, respectively).

**Results:**

There were no significant differences in the SE of MR, Sim K and UDVA between the 6.0 OZ group and the 6.5 OZ group over 1 year of follow-up after PRK, and the 6.0 OZ group required less ΔTCT/CD than the 6.5 OZ group. The 6.5 OZ group showed better results in terms of post-operative HOAs of RMS, SA and Q value. When comparing that pattern of change in Sim K, there was no significant difference between the 6.0 OZ group and the 6.5 OZ group.

**Conclusions:**

The clinical refractive outcomes and regression after PRK using Mel 90 excimer laser with a 6.0 OZ were comparable to those with a 6.5 OZ.

## Background

There has been rapid development in laser surgery for myopia and myopic astigmatism. Photorefractive keratectomy (PRK) was introduced in 1983, and has become a safe and effective method for treating myopia [1–4]. The Mel 90 excimer laser with the advanced ablation algorithm (Triple-A) profile, developed by Carl Zeiss Meditec AG, was reported to achieve better visual acuity outcomes with lower ablation depth, and has frequencies up to 500 Hz with better a tracking system than previous excimer lasers [5]. Further optical design has been customized to reduce spherical aberration and irregular astigmatism [6–8].

Previous studies suggest that a larger optical zone may improve optical quality and stability. Historically the optimal optical zone for myopic PRK was smaller than 5.0 mm, but it has increased up to 6.0 mm – 6.5 mm recently because small optical zones induced glare and halo symptoms in scotopic conditions [9–12], and patients with a larger optical zone are predicted to have less initial overcorrection and less myopic regression [13, 14]. However, there is need for limiting ablation diameter in that a larger ablation diameter requires greater ablation depth [4]. Minimizing ablation depth may reduce adverse effects on postoperative wound healing and haze [9, 15–17], although there are conflicting studies [11, 14]. A larger optical zone can lead to decreased biomechanical stability causing keratoectasia [14, 18].

Recent studies have reported various clinical outcomes after laser refractive surgery with a 6.5 mm optical zone [5, 19–21], and one study compared a 6.0 mm optical zone with a 6.5 mm optical zone in terms of aberration values after PRK. In that study, the 6.5 mm optical zone group had a lower root mean square of higher order aberrations (RMS of HOAs) than the 6.0-mm optical zone group until 3 months postoperatively [22]. However, there are not many studies comparing a 6.0 mm optical zone with a 6.5 mm optical zone after laser refractive surgery over a long-term follow up period. In this study, we assessed the changes in clinical outcomes and the degree of regression after PRK between a 6.0 mm optical zone group and a 6.5 mm optical zone group over 1 year of follow-up.

## Methods

We retrospectively reviewed the medical records of 95 eyes of 48 patients that undergone PRK with a 6.0 mm optical zone (*n* = 40) or a 6.5 mm optical zone (*n* = 55) for myopia and myopic astigmatism in the Ophthalmology Department at Kangbuk Samsung Hospital, Seoul, Korea from June 2014 to December 2017 and had completed over 12 months of postoperative follow-up. The study was approved by the ethics committee of the local institutional review board.-.

PRK was performed by one surgeon (CY Choi). None of the patients had a history of ocular surgery, signs of corneal disease, corneal opacity, glaucoma, connective tissue disorder or untreated retinal disease. Data including spherical equivalent of manifest refraction (SE of MR), Sim K, thinnest corneal thickness, change in thinnest corneal thickness (initial value divided by the corrected diopter [ΔTCT/CD]), Q value, corneal HOAs and spherical aberration (SA) pre-operatively, at 3 and 6 months postoperatively and at the last follow-up visit were collected to evaluate optical performance, quality and regression.

### Preoperative evaluation

Preoperative ophthalmic evaluations corrected distance visual acuity (CDVA), intraocular pressure, manifest and cycloplegic refraction, slit lamp examination, fundus examination, specular microscopy (NSP-9900; Konan Medical, Inc., Hyogo, Japan), corneal pachymeter (Sonoscan 4000AP; Sonomed Inc., New York, NY), topography (Galilei G4; Zimmer Ophthalmics, Port, Switzerland) and wavefront analysis (KR-1 W; Topcon, Tokyo, Japan).

### PRK

Local anesthesia was achieved with topical proparacaine hydrochloride 0.5% solution drops (Alcane; Alcon, Fort Worth, TX). The surgeon removed the epithelium using a blunt spatula (K2–3700; Katena Products, Inc., Denville, NJ). Laser ablation was conducted with the Mel 90 excimer laser (Carl Zeiss Meditec, Jena, Germany, Triple-A profile). A sponge soaked in mitomycin-C (MMC) 0.02% was applied on the stromal bed for 10 s followed by balanced sterile solution irrigation and bandaging with a contact lens (Acuvue Oasys, Johnson & Johnson Vision Care, Inc., Jacksonville, FL). The target refraction was plano for all patients.

### Post-operative management and evaluation

Topical levofloxacin 0.5% solution (Cravit; Santen, Japan) and fluorometholone 0.1% solution (Ocumetholone; Samil, Korea) were started immediately after the surgical procedure. The eye drops were applied four times daily for 1 week, two times daily for 1 month and finally once a day for another 2 months. Routine post-operative follow up visits were scheduled at 1 and 4 days, 1 week, and 1, 3, and 6 months, 1 year and every year thereafter. Uncorrected distance visual acuity (UDVA) was assessed, and manifest refraction, corneal topography, wavefront analysis and slit lamp biomicroscopy were performed at each visit.

### Statistical analysis

Statistical analyses were performed using SPSS software (version 24.0; SPSS Inc.). The mean ± standard deviation (SD) was calculated. Data were first assessed for a normal distribution. In cases of normal distribution, group comparisons were made using an independent two-sample t-test and two-way repeated measures analysis of variance (ANOVA). A minimum sample size of 21 eyes per group was necessary to detect a difference in clinical outcomes between groups to achieve 80% statistical power and 0.05 probability using a two-sided two-sample unequal-variance t-test. The magnitude of the effect chosen was the significant difference previously found when comparing a 6.0 mm optical zone and a 6.5 mm optical zone with the largest required sample size [22]. One-way repeated measures ANOVA with Bonferroni multiple comparisons was performed to determine the difference between data from post-operative visits at 3 and 6 months and from the last follow-up visit for each group. The Mann-Whitney U test and Wilcoxon signed ranks test were used for continuous data that did not approximate a normal distribution. A *P*-value less than 0.05 was considered significant.

## Results

The sample included 95 eyes from 48 patients. Among the patients, 20 patients of 6.0 OZ group and 27 patients of 6.5 OZ group were bilateral cases, and one patient of 6.5 OZ group was a unilateral case. The last follow-up visit was done after 12 months postoperatively in both groups, and the longest follow-up period was 38 months in the 6.0 OZ group and 30 months in the 6.5 OZ group. There were no significant differences in mean age of subjects and preoperative manifest refractions, Sim K, thinnest pachymetry, Q value, RMS of HOAs, or spherical aberration between the two groups (Table 1). Supplementary figures show nine standard graphs for reporting post-operative refractive surgery outcomes for each 6.0 OZ group and 6.5 OZ group (Supplementary Fig. 1, 2).

**Table 1:** Demographics and Preoperative Parameters

Parameters	Demographics		*p* value
	Group 1 (Mean ± SD)	Group 2 (Mean ± SD)	
Age (years)	29.92 ± 3.92	28.41 ± 6.09	0.165
Sex (M:F)	9:11	12:16	–
Follow up period (months)	20.71 ± 10.52	17.47 ± 6.57	0.07
SE of MR (D)	−6.31 ± 2.43	−5.47 ± 2.15	0.078
Sim K (D)^a^	43.53 ± 1.40	43.09 ± 0.92	0.072
TCT (μm)^a^	545.71 ± 27.30	555.67 ± 33.25	0.131
Q value^a^	0.14 ± 0.12	0.16 ± 0.13	0.368
RMS of HOAs (μm)^b^	0.38 ± 0.11	0.36 ± 0.10	0.293
SA (μm)^b^	0.22 ± 0.08	0.21 ± 0.07	0.300
Number of eyes	40	55	–

*SD* Standard deviation, *SE of MR* Spherical equivalent of manifest refraction, *Sim K* Simulated keratometry, *TCT* Thinnest corneal thickness, *RMS of HOAs* Route mean square of higher order abberations, *SA* Spherical aberration

^a^All topographic parameters were measured by Galilei G4 (Sim K was measured in the 4.0 mm zone, TCT was measured on the thinnest pachy map, Q value was measured in the 8.0 mm zone)

^b^All aberration parameters were measured by KR-1 W (RMS of HOAs and SA)

**Fig. 1:**
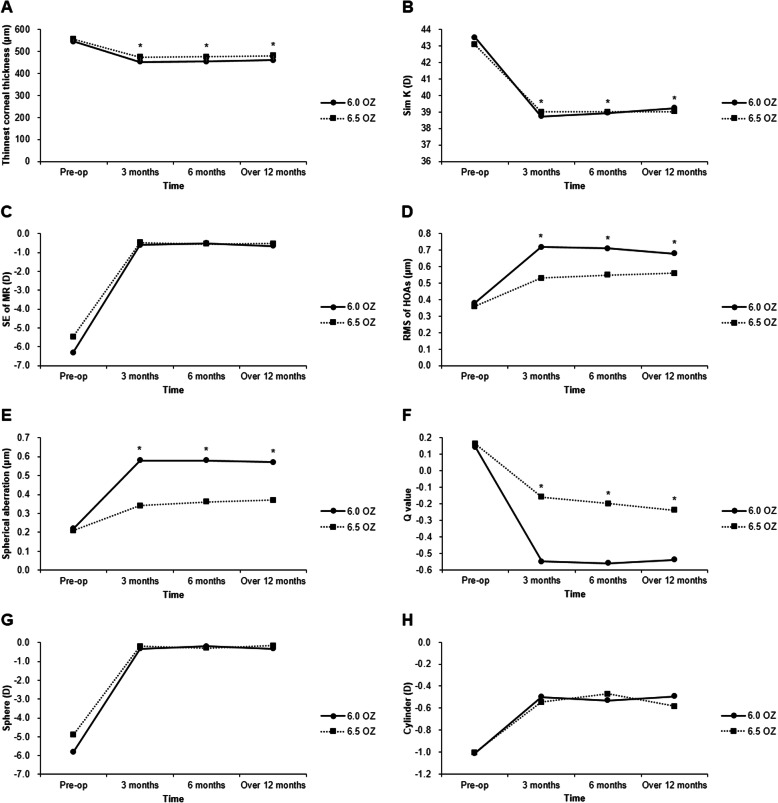
Postoperative changes of the (**a**) thinnest corneal thickness, (**b**) simulated keratometry (SimK), (**c**) spherical equivalent of manifest refraction (SE of MR), (**d**) route mean square of higher order aberrations (RMS of HOAs), (**e**) spherical aberration, (**f**) Q value, (**g**) sphere and (**h**) cylinder. Asterisks indicate a significant difference at a <  0.05

The Sim K values after PRK showed a significant increase in Sim K from the six-month visit to the last visit in the 6.0 OZ group only (*p* <  0.001). However, there was no significant interaction between optical zone size and the change in pattern by time (*p* = 0.118, RM ANOVA), and there was not a significant difference in Sim K between the 6.0 OZ group and the 6.5 OZ group at each of the follow-up visits (3 months, *p* = 0.594, 6 months, *p* = 0.907, last follow up, *p* = 0.667) (Table 2, Fig. 1).

**Table 2:** Changes in Parameters After Photorefractive Keratectomy and Comparisons Between the 6.0 mm Optical Zone and 6.5 mm Optical Zone Groups

Parameters^a^	3 months (Mean ± SD)	6 months (Mean ± SD)	Last visit (Mean ± SD)	*p* value^*^	
				3–6 months	6–over 12 months
TCT (μm)					
6.0 OZ	452.81 ± 51.03	454.69 ± 52.18	460.06 ± 49.71	0.851	< 0.001^†^
6.5 OZ	474.04 ± 36.91	476.78 ± 35.67	480.75 ± 35.49	0.057	< 0.001^†^
*p* value	0.035	0.030	0.035		
ΔTCT/CD (μm/D)					
6.0 OZ	12.76 ± 5.13	12.38 ± 5.63	11.62 ± 5.98	0.061	0.001^†^
6.5 OZ	15.02 ± 3.89	14.61 ± 4.07	13.80 ± 4.12	0.058	< 0.001^†^
*p* value	0.018	0.043	0.024		
Sim K (D)				0.118^**^	
6.0 OZ	38.73 ± 2.58	38.94 ± 2.31	39.24 ± 2.26		
6.5 OZ	39.01 ± 2.29	39.00 ± 2.27	39.03 ± 2.17		
*p* value	0.594	0.907	0.667		
SE of MR (D)					
6.0 OZ	−0.61 ± 0.57	−0.52 ± 0.62	− 0.66 ± 0.50	0.968	0.072
6.5 OZ	− 0.47 ± 0.45	−0.55 ± 0.57	−0.54 ± 0.42	0.474	0.660
*p* value	0.181	0.740	0.216		
Sph (D)					
6.0 OZ	−0.33 ± 0.50	−0.20 ± 0.52	−0.33 ± 0.47	0.247	0.202
6.5 OZ	−0.20 ± 0.50	−0.28 ± 0.63	−0.15 ± 0.63	0.309	0.443
*p* value	0.252	0.630	0.220		
Cyl (D)					
6.0 OZ	−0.50 ± 0.29	−0.53 ± 0.27	−0.49 ± 0.25	0.475	0.156
6.5 OZ	−0.55 ± 0.38	−0.47 ± 0.42	−0.58 ± 0.43	0.186	0.098
*p* value	0.788	0.173	0.191		
RMS of HOAs (μm)					
6.0 OZ	0.72 ± 0.22	0.71 ± 0.22	0.68 ± 0.19	0.962	0.043
6.5 OZ	0.53 ± 0.21	0.55 ± 0.23	0.56 ± 0.20	0.400	0.236
*p* value	< 0.001	0.001	0.005		
SA (μm)					
6.0 OZ	0.58 ± 0.20	0.58 ± 0.21	0.57 ± 0.17	0.132	0.663
6.5 OZ	0.34 ± 0.15	0.36 ± 0.14	0.37 ± 0.13	0.667	0.137
*p* value	< 0.001	< 0.001	< 0.001		
Q value					
6.0 OZ	−0.55 ± 0.43	−0.56 ± 0.36	−0.54 ± 0.33	0.590	0.079
6.5 OZ	−0.16 ± 0.32	−0.20 ± 0.32	−0.24 ± 0.33	0.496	0.148
*p* value	< 0.001	< 0.001	< 0.001		
UDVA (logMAR)					
6.0 OZ	−0.17 ± 0.08	−0.17 ± 0.09	−0.16 ± 0.10	10.000	0.403
6.5 OZ	−0.17 ± 0.10	−0.18 ± 0.09	−0.17 ± 0.09	0.637	0.763
*p* value	0.673	0.739	0.538		

*SD* Standard deviation, *Sim K* Simulated keratometry, *TCT* Thinnest corneal thickness, *SE of MR* Spherical equivalent of manifest refraction, *RMS of HOAs* Route mean square of higher order abberations, *SA* Spherical aberration, *UDVA* Uncorrected distance visual acuity, *logMAR* Logarithm of the minimum angle of resolution

^†^Significant difference at α < 0.05

^*^Comparison of parameters between 6 months and beyond 12 months postoperatively. There was no significant difference in any parameter between three months and six months postoperatively

^**^Sim K was normally distributed, thus the intergroup comparisons at three months, six months, and the last follow-up visit were made using the independent two samples t-test and comparisons in patterns of change by time between 6.0 OZ and 6.5 OZ were analyzed by two-way repeated measures ANOVA

^a^None of the parameters except Sim K were normally distributed, so intergroup comparisons were done using the Mann-Whitney U test, and the intragroup changes between 3 and 6 months and 6 months and the last visit were analyzed by the Wilcoxon signed ranks test

There were no significant differences in SE of MR values between the 6.0 OZ group and the 6.5 OZ group at any of the follow-up visits (3 months, *p* = 0.181, 6 months, *p* = 0.740, last follow up, *p* = 0.216), and there were no significant differences from 3 months to 6 months postoperatively (6.0 OZ, *p* = 0.968; 6.5 OZ, *p* = 0.474) or from 6 months postoperatively to the last visit (6.0 OZ, *p* = 0.115; 6.5 OZ, *p* = 0.660) (Table 2, Fig. 1). The residual sphere and cylinder were not significantly different between groups at any of the post-operative visits (Sphere: 3 months, *p* = 0.252, 6 months, *p* = 0.630, last follow up, *p* = 0.220; Cylinder: 3 months, *p* = 0.788, 6 months, *p* = 0.173, last follow up, *p* = 0.191) nor were there any intragroup changes between postoperative visits (Sphere: 3–6 months: 6.0 OZ, *p* = 0.247, 6.5 OZ, *p* = 0.309, 6 months-last visit: 6.0 OZ, *p* = 0.202, 6.5 OZ, *p* = 0.443; Cylinder: 3–6 months: 6.0 OZ, *p* = 0.475, 6.5 OZ, *p* = 0.186, 6 months-last visit: 6.0 OZ, *p* = 0.156, 6.5 OZ, *p* = 0.098) (Table 2).

The thinnest corneal thickness increased from 6 months post-operatively to the last visit (6.0 OZ, *p* <  0.001; 6.5 OZ, *p* <  0.001), but there was no significant change between three and six months post-operatively (6.0 OZ, *p* = 0.851; 6.5 OZ, *p* = 0.057). The thinnest corneal thickness after PRK was thinner in the 6.0 OZ group than that in the 6.5 OZ group across all of the follow-up visits (3 months, *p* = 0.035, 6 months, *p* = 0.03, last follow up, *p* = 0.035). However, the thinnest corneal thickness after PRK depended on the amount of correction, thus the changes in thinnest corneal thickness from pre-operative values were divided by the corrected diopter (ΔTCT/CD) to adjust ablation depth by optical zone. Significant differences in ΔTCT/CD were found from the 6-month to the last visit in the 6.0 OZ group (*p* = 0.001) and the 6.5 OZ group (*p* <  0.001), but not from the 3-month to 6-month visits (6.0 OZ, *p* = 0.061; 6.5 OZ, *p* = 0.058). The 6.5 OZ group had a significantly greater ΔTCT/CD than the 6.0 OZ group at each follow-up visit (3 months, *p* = 0.018, 6 months, *p* = 0.043, last follow up, *p* = 0.024) (Table 2, Fig. 1).

The 6.5 OZ group showed significantly lower RMS of HOAs than the 6.0 OZ group at each follow up visit (3 months, *p* <  0.001, 6 months, *p* = 0.001, last follow up, *p* = 0.005). Significant differences in the RMS of HOAs were found from the six-month to the last visit in the 6.0 OZ group only (*p* = 0.043). The 6.5 OZ group had a significantly lower SA than the 6.0 OZ group at each follow up visit (3 months, *p* <  0.001, 6 months, *p* <  0.001, last follow up, *p* <  0.001). There were no significant differences from 3 months to 6 months post-operatively (6.0 OZ, *p* = 0.132; 6.5 OZ, *p* = 0.667), or from 6 months post-operatively to the last visit (6.0 OZ, *p* = 0.191; 6.5 OZ, *p* = 0.137) (Table 2, Fig. 1).

The Q value was significantly lower in the 6.5 OZ group than that in 6.0 OZ group at each follow up visit (3 months, *p* <  0.001, 6 months, *p* <  0.001, last follow up, *p* <  0.001). There were no significant differences from three months to six months post-operatively (6.0 OZ, *p* = 0.590; 6.5 OZ, *p* = 0.496), or from 6 months post-operatively to the last visit (6.0 OZ, *p* = 0.079; 6.5 OZ, *p* = 0.148) (Table 2, Fig. 1).

## Discussion

Current laser ablation techniques have developed with an increase in frequency and improved tracking system enabling more precise corneal surface remodeling [5]. Furthermore, customized optic design has reduced postoperative mechanical complications and pain, and yielded faster recovery times with better clinical outcomes [6, 8, 23]. The newer excimer lasers with advanced profile have allowed surgeons to perform surface ablation surgeries with a larger optical zone by enhanced projection error compensation [24].

Previous studies have shown advantages of a larger optical zone for laser refractive surgery. Kim et al. [9] reported more night vision problems and ablation decentration with a 5.0 mm optical zone than with a 6.0 mm optical zone. The study by O’Brart et al. [14] showed less initial overcorrection and regression with a 6.0 mm optical zone than with a 5.0 mm optical zone over 6 months of follow-up. Rajan et al. [13] reported a larger optical zone had better results in terms of early hyperopic shift, regression, and night haloes for patients who had PRK with 4.0 mm optical zone, 5.0 mm optical zone, and 6.0 mm optical. We compared a 6.0 mm optical zone and the larger optical zone (6.5 mm) using more clinical factors and found no significant differences between the groups. This result may due to the fact that we used the newer excimer laser with a more advanced ablation profile than previous studies.

Seo et al. [22] compared aberration factors after PRK with 6.0 mm optical zone and 6.5 mm optical zone. In their study, RMS of HOAs in the larger zone (6.5 mm) group were less than those in the 6.0 mm optical zone group, and both groups showed a significant increase in SA from preoperatively to 3 months postoperatively. In the present study, we found that the RMS of HOAs and SA in the 6.5 OZ group were lower than in the 6.0 OZ group at 3, 6, and over 12 months after surgery which was longer follow-up period than the previous study of Seo et al. [22]. HOA wavefront of both groups was measured under the same condition by using TAPCON regardless of the pupil size. We also focused on intragroup changes after surgery, and found that there was a decrease in the RMS of HOAs from 6 months to beyond 12 months post-operatively in the 6.0 OZ group, but not in the 6.5 OZ group, and no significant changes in SA after 3 months post-operatively in either group.

Based on early clinical studies of PRK, a deeper ablation depth was thought to increase corneal haze [3, 17]. However, Rajan et al. [13] reported a larger zone yielded less corneal haze, and recent studies pointed out that a wound edge with a larger ablation zone had a more gradual slope, which enabled smooth epithelial migration and minimized hyperplasia and corneal haze [25, 26]. In terms of biomechanical stability, reducing residual bed thickness is related to the weakening of cohesive tensile strength, tangential tensile strength and shear strength [27–29], which is more likely to result in mechanical postoperative adverse effects such as keratoectasis [14, 18, 30]. Therefore, when surgeons set the optical zone for PRK, there should be a limitation on optical zone size based on biomechanical stability and other individual factors such as refractive error, pupil size and age.

Manifest refraction is considered a clinically significant factor influencing patient satisfaction after PRK. In our study, there was no significant regressive change in either the 6.0 OZ group or 6.5 OZ group, and no significant differences between the two groups at each visit. This was consistent with the outcomes for UDVA in the 6.0 OZ group and 6.5 OZ group.

Sim K in the 6.0 OZ group increased significantly from six months to beyond twelve months postoperatively in this study (by paired t-test). However, the difference was 0.3 D, which is needed to be assessed through a survey for measuring the discomfort in daily life in the further study, and we found that the regression patterns from preoperative visit to any postoperative visit for the 6.0 OZ group and 6.5 OZ group were not significant (*p* = 0.118, 2 way repeated measured ANOVA). Therefore, there was no significant clinical regression in Sim K in either group.

On the other hand, the RMS of HOAs, SA, and Q value were lower in the 6.5 OZ group than those in the 6.0 OZ group, which meant PRK with 6.5 OZ might yield better visual quality. Corneal haze with epithelial hyperplasia and stromal remodeling can occur after laser refractive surgery [31], and this thickening can be evident up to 12 months after PRK [31–34], which makes clinical outcomes measured in this period unstable. In this study, the last visits were at least 12 months postoperatively, thus PRK with 6.5 mm optical zone showed lower aberration values than PRK with 6.0 mm zone even in the long term.

The RMS of HOAs showed significant changes between 6 months and beyond 12 months postoperatively likely because it is more reflective of the numerous Zernike’s coefficients (SA, Coma, trefoil, etc.), but SA is more a clinically interesting factor generally caused after myopic and hyperopic correction, and it might be related to variety of visual symptoms. Moreover, there is individual variation in neural transfer function, and common clinical measures of visual function are not sensitive at low levels of aberration [35–37]. Therefore, better aberration values are not necessarily correlated with improved visual performance.

The thinnest corneal thickness in this study increased significantly until the last follow up visit, but the differences ranged from 3 to 6 μm, which is required to evaluate and conduct surveys prospectively, in order to see if these differences are clinically significant or not in their daily activity. The differences may be due to measurement error and/or intra-examination error in thinnest pachymetry, depending on which point of the cornea was selected for measurement. Results would be more reliable if the thinnest corneal thickness was measured at a designated point with ultrasound repeatedly at every visit.

To evaluate the regressive pattern after laser refractive surgery precisely, it requires corneal epithelial and stromal map analysis using optical coherence tomography (OCT) [38]. However, this study was proceeded retrospectively, where we had to estimate the regression based on the routine follow-up exams that were conducted after PRK, therefore we had to rely on the corneal thickness map of corneal topography instead of OCT, thus further study is required to measure corneal epithelial and stromal change to estimate regressive pattern following PRK.

In this study, we performed PRK using the Mel 90 excimer laser with the Triple-A profile which is improved from previous ASA and TSA profiles in that it has better error compensation function and target asphericity control, which minimizes ablation depth, adjusts for spherical aberration and prevents myopic regression [39]. The surgeon can also choose a frequency up to 500 Hz across the surgery in Mel 90 excimer laser, which enables faster ablation time for correction [39, 40]. Thus, clinical outcomes after PRK with the same optical zone size can differ according to which excimer laser used, and it therefore seems inappropriate to compare this study with clinical data from studies that used the previous version of the excimer laser for PRK in the same way.

Previous long-term follow up studies after PRK were up to 12 years [13]. However, that study compared different optical zones (4.0 mm, 5.0 mm, 6.0 mm), and in our study (6.0 mm, 6.5 mm) we used a different excimer laser and ablation profile. A longer study seems necessary in that we noted a significant increase in the thinnest corneal thickness from 6 months postoperatively to beyond 12 months, although this study had the longest follow-up period of all clinical studies after PRK with the Mel 90 excimer laser to date.

## Conclusion

The 6.5 OZ group had better RMS of HOAs, SA and Q value during the follow-up period in this study. However, it is questionable whether a difference in wave front index could lead to optical performance improvement. We found that there were no significant differences in SE of MR, Sim K, UDVA, or regression patterns between groups. In other words, clinical outcomes and regression after PRK with a 6.0 mm optical zone was comparable to those after PRK with a 6.5 mm optical zone.

The optical zone selected for refractive surgery depends on individual refraction and keratometer profile, and biomechanical safety, which is based on several factors including total corneal thickness, residual bed thickness and ablation design. Therefore, further studies and effort are necessary for clinical ophthalmologists to compare between 6.0 mm optical zone and 6.5 mm optical zone, and additional studies of the 6.3 mm optical zone are required to better identify optimal optical zones.

## Supplementary information

**Additional file 1.**

**Additional file 2.**

## Data Availability

The datasets used and/or analyzed during the current study available from the corresponding author on reasonable request.

## Abbreviations

CDVA: Corrected distance visual acuity
HOAs: Higher order aberrations
MMC: Mitomycin-C
MR: Manifest refraction
PRK: Photorefractive keratectomy
RMS: Root mean square
SA: Spherical aberration
SE: Spherical equivalent
SD: Standard deviation
Sim K: Simulated keratometry
UDVA: Uncorrected distance visual acuity
