# Supplementary figures and images for: Comparison of refractive outcomes after photorefractive keratectomy with different optical zones using Mel 90 excimer laser
Source: BMC Ophthalmol. 2020 Jul 9;20:270. doi: 10.1186/s12886-020-01537-3 (PMC7346386; doi:10.1186/s12886-020-01537-3)

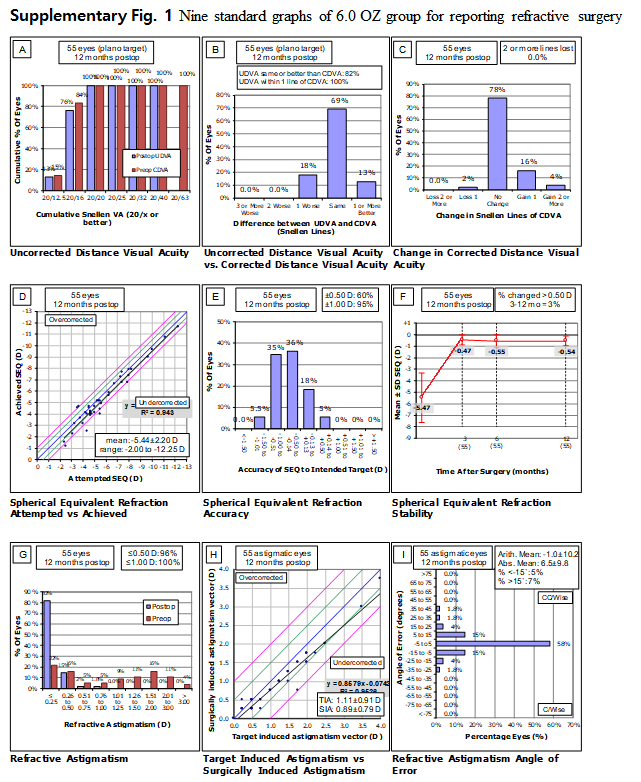

Supplement: Supplementary file 1 — Additional file 1. [file 12886_2020_1537_MOESM1_ESM.tif]

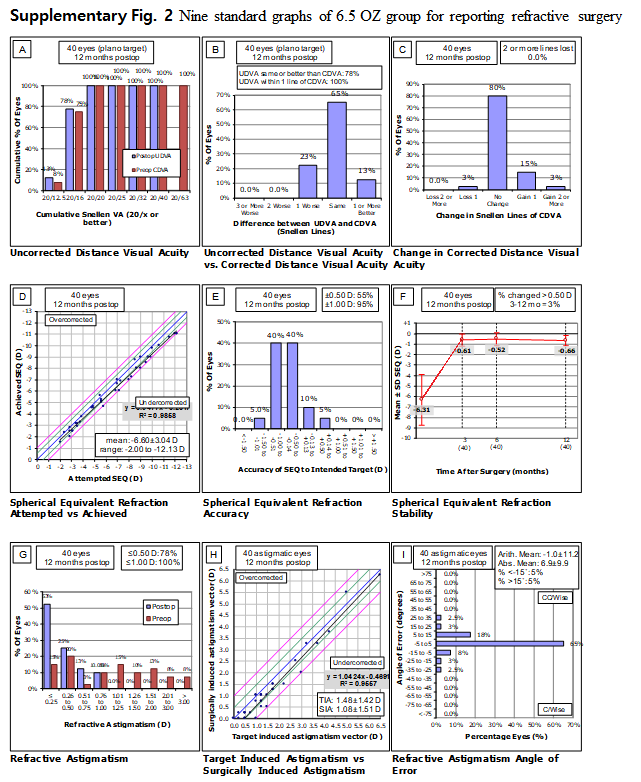

Supplement: Supplementary file 2 — Additional file 2. [file 12886_2020_1537_MOESM2_ESM.tif]
